# Oxidative Stress and Semen Quality Among Night- and Day-Shift Workers: A Cross-Sectional Study

**DOI:** 10.3390/antiox14070802

**Published:** 2025-06-28

**Authors:** Luca Boeri, Federica Passarelli, Ludovico Maria Basadonna, Edoardo Sorba, Giorgio Graps, Fabio Ciamarra, Damiano Dagnino, Valentina Parolin, Marco Nizzardo, Gianpaolo Lucignani, Emanuele Montanari

**Affiliations:** 1 Department of Urology, IRCCS Fondazione Ca’ Granda, Policlinico di Milano, 20122 Milan, Italy; federica.passarelli@unimi.it (F.P.); ludovico.basadonna@unimi.it (L.M.B.); edoardo.sorba@unimi.it (E.S.); giorgio.graps@unimi.it (G.G.); fabio.ciamarra@unimi.it (F.C.); damiano.dagnino@unimi.it (D.D.); valentina.parolin@unimi.it (V.P.); marco.nizzardo@unimi.it (M.N.); gianpaolo.lucignani@unimi.it (G.L.); emanuele.montanari@policlinico.mi.it (E.M.); 2 Dipartimento di Scienze Cliniche e di Comunità, Dipartimento di Eccellenza 2023-2027, Università degli studi di Milano, 20121 Milan, Italy

**Keywords:** night-shift worker, infertility, sperm quality, antioxidant, oxidative stress

## Abstract

**Introduction:** Infertility affects 15% of couples, with oxidative stress recognized as a key contributor to male infertility. Night-shift work, through circadian disruption, may exacerbate oxidative imbalance and impair reproductive function. This study investigates the impact of night-shift work on oxidative stress and semen quality and evaluates the potential benefits of antioxidant supplementation in this context. **Materials and Methods:** We retrospectively analysed 96 white-European men aged 18–45, seeking fertility assessment at a single academic centre. Participants were classified as day or night workers based on their shift schedule, and all underwent standardised clinical, hormonal, and semen evaluations. Oxidative stress was assessed using the d-ROMs test. A subgroup of 40 patients (20 per group) treated for 3 months with antioxidant supplementation (Drolessano) to evaluate changes in oxidative stress and semen parameters was also considered. Statistical comparisons were performed using non-parametric tests and logistic regression analyses. **Results:** Night-shift workers exhibit significantly higher oxidative stress levels compared to day workers (median D-ROMs values of 340 vs. 280 U.CARR, *p* = 0.01), and a greater proportion of men exceeding the oxidative stress threshold (74.4% vs. 24.4%, *p* = 0.01). Logistic regression confirmed night-shift work as an independent predictor of elevated oxidative stress (OR 2.1, *p* = 0.001), even after adjusting for age and smoking. Following three months of antioxidant supplementation with Drolessano, both groups experienced significant reductions in oxidative stress (all *p* < 0.01), but night workers showed a substantially greater decrease (mean change −58.5 vs. −15.4 U.CARR, *p* = 0.001). Improvements in semen quality, including sperm concentration, motility, and morphology, were also more pronounced in the night group after treatment. **Conclusions:** At baseline, night-shift workers had significantly higher oxidative stress than day workers, likely due to circadian disruption. Both groups improved after antioxidant treatment, but night workers showed a greater reduction in D-ROMs. This pilot study might suggest a potential benefit of antioxidant therapy particularly in night workers.

## 1. Introduction

Infertility is defined as the failure of a sexually active couple, not using contraception, to conceive naturally within 12 months [1]. It represent a major global health issue, affecting approximately 15% of couples of reproductive age, with male factors contributing to nearly half of all cases [2].

A wide range of conditions have been implicated in male infertility, including genetic abnormalities, endocrine disorders, infections, varicocele, and exposure to environmental and lifestyle-related stressors. Among these, oxidative stress has emerged as a central mechanism linking various risk factors to impaired sperm function [1]. Oxidative stress describes a pathological state in which an imbalance between the production of reactive oxygen species (ROS), highly reactive molecules derived from oxygen, and the body’s antioxidant defences leads to cellular injury [3]. Under normal conditions, excess ROS generated by physiological immune responses, hypoxia, and exposure to oxidizing agents are neutralized by enzymatic and non-enzymatic antioxidant systems, preserving redox balance and protecting cells from damage [4]. Spermatozoa, however, possess minimal intrinsic antioxidant ability and a membrane rich in polyunsaturated fatty acids, making them especially susceptible to oxidative damage [3]. Indeed, oxidative stress has emerged as a pivotal pathogenic mechanism in the context of male reproductive health [5].

Oxidative stress is estimated to underlie 30–80% of subfertility cases, leading to the conceptualization of a specific category: male oxidative stress infertility (MOSI) [6]. As a response, several studies have explored the therapeutic potential of antioxidant supplementation, demonstrating improvements in sperm quality parameters [7]. Nevertheless, due to the still incomplete understanding of MOSI’s underlying causes, no standardized treatment guidelines have yet been established.

In recent years, attention has increasingly turned to the impact of circadian rhythm disruption, particularly among night-shift workers, as a potential but underexplored contributor to male infertility. In modern industrial societies, night-shift work has become commonplace due to the increasing demand for continuous 24/7 services and production. However, this form of employment poses substantial physiological challenges. Human reproductive processes, including testosterone secretion, spermatogenesis, and sexual behaviour, are regulated by the circadian system, which is synchronized primarily by the light-dark cycle [8,9].

Disruption of circadian rhythms, caused by exposure to artificial light at night, irregular sleep patterns, and dysregulated food intake, has been shown to alter hormonal profiles, including testosterone, melatonin, and cortisol, impair sleep quality, and increase systemic oxidative stress [10]. The mechanisms behind the observed oxidative imbalance in night workers may involve prolonged wakefulness and increased mitochondrial activity, which elevates ROS production. Furthermore, suppression of melatonin, an endogenous antioxidant, due to nocturnal light exposure likely exacerbates oxidative stress [11].

Epidemiological studies have linked night-shift work to a range of adverse health outcomes, including metabolic syndrome, cardiovascular disease, neurodegenerative conditions, and cancer. Many of these conditions share oxidative stress and inflammation as common underlying mechanisms [12]. Emerging evidence also suggests that night-shift work may negatively affect male reproductive health, with reports of altered semen quality, increased sperm DNA fragmentation, and subfertility among shift-working men compared to those with regular daytime schedules [13,14].

Given the potentially synergistic effects of circadian misalignment and oxidative stress on male reproductive function, there is a compelling need to identify and implement effective therapeutic strategies capable of counteracting its harmful effects. Indeed, while oxidative stress has been broadly studied in the context of male infertility, few studies have specifically addressed the combined impact of circadian disruption and oxidative imbalance on semen quality. Moreover, previous research has not comprehensively evaluated antioxidant interventions in shift-working populations. Therefore, the primary aim of the present study was to investigate the impact of night-shift work on oxidative status and semen quality in men seeking medical attention for fertility evaluation. As a secondary aim, we conducted a pilot study to provide preliminary evidence on the potential effects of antioxidant supplementation on semen oxidative stress and quality in night versus day workers.

## 2. Materials and Methods

We retrospectively analysed data collected from a cohort of 114 white-European men, aged 18–45, seeking first medical help, at a single academic centre, for fertility evaluation between September 2023 and December 2024. Based on the WHO definition of couple’s infertility, defined as not conceiving a pregnancy after at least 12 months of unprotected intercourses regardless of whether or not a pregnancy ultimately occurs, all participants were not part of infertile couples [15].

All participants were homogenously assessed by the same expert academic urologist (L.B.), with a thorough medical history and a complete physical examination [1]. Health-significant comorbidities were scored with the Charlson Comorbidity Index (CCI) [16,17]. Likewise, weight and height were measured, calculating body mass index (BMI) for each participant [18]. Testes volume (TV) was assessed in all cases using Prader’s orchidometer estimation [19]; for the specific purpose of this study, we calculated the mean value between the two sides. Varicocele was also clinically assessed in every patient. Cigarette smoking status was investigated and categorized as former/no smoker vs. current smoker [20]. Working-shift plan was also inquired. All participants worked in the same routine for at least six months in administrative and health-related functions (nurse, physiotherapist, physicians, laboratory technician) and were classified according to their work shift as (1) day workers (D group), who worked only during the day, morning and/or afternoon, without developing any work activity at night (if these workers had an extra shift, this occurred only during the day); and (2) night workers (N group), who worked at least six hours after midnight, with and without daytime additional work activities. Only workers who perform at least six night shifts per month were considered in Group N.

Venous blood samples were drawn from each patient between 7 a.m. and 11 a.m. after an overnight fast. Follicle-stimulating hormone (FSH), luteinizing hormone (LH), total testosterone (tT), and prolactin levels were measured for every individual. Moreover, reactive oxygen metabolites were measured using the derivatives of reactive oxidative metabolites (d-ROMs) test (DIACRON INTERNATIONAL, Grosseto, Italy), with reference values of 250–300 U.CARR and intra-/inter-assay CVs of 0.3–6.6% and 0.3–5.1%, respectively [21]

At baseline, all patients underwent two consecutive semen analyses, analysed according to WHO criteria [22,23]. For the specific purposes of this study, we considered semen volume, sperm concentration, progressive sperm motility, and normal morphology. The same laboratory was used for analyses of all parameters.

Semen samples were collected in the laboratory by masturbation after a sexual abstinence of 2–5 days. Thereafter, samples were analysed within 30 min of ejaculation, in accordance with the WHO criteria [23]. The improved Neubauer hemocytometer chamber (100-μm-deep; Brand™ Blaubrand™ Neubauer Improved Counting Chambers, Fisher Scientific, Loughborough, UK) was used to calculate sperm concentration and total sperm count in the ejaculate. Sperm morphology was assessed through the following steps: preparation of a smear of semen on a slide; fixing and staining the slide (Testsimplets1Prestained Slides, Waldeck GmbH & Co. KG, Münster, Germany); examination with brightfield optics at ×1000 magnification (Nikon Eclipse E 200, Nikon Instruments Europe B.V., Rome, Italy) with oil immersion; and assessment of approximately 200 spermatozoa per replicate for the percentage of normal or abnormal forms. Sperm motility was assessed by mixing twice the sample, using a wet preparation of 20.7 microM deep for each replicate, by examining the slide with phase-contrast optics at ×200 magnification and by assessing approximately 200 spermatozoa per replicate for the percentage of different motile categories.

We excluded patients that had pre-existing or concurrent diseases that may have influenced oxidative stress levels (*n* = 10); obesity (*n* = 7); had a different work shift from the two classifications of this study (*n* = 8). A final cohort of 96 participants was considered for the primary aim of the study.

For the secondary aim, we investigated a subgroup of the study population, forty workers (20 night and 20 day workers), that underwent a 3-month trial involving antioxidant therapy to evaluate its potential effects on oxidative stress parameters. Participants were treated with a dietary supplement containing solanum lycopersicum (100 mg), brassica oleracea (333 mg), silybum marianum (50 mg), glutathione (50 mg), aesculus hippocastanum (2 mg), tryptophane (25 mg), camellia sinensis (25 mg) (namely Drolessano). Drolessano is an antioxidant supplement used in clinical practice to counteract oxidative stress. Participants were instructed to take one tablet daily in the morning for three months. All participants were naïve for other antioxidant treatments and performed their first semen evaluation during the study period.

The assessment visit was performed in person after 3 months of Drolessano treatment. At follow-up, semen parameters and D-ROMS were recorded.

After treatment we also evaluated the change from baseline to follow-up in terms of conventional semen characteristics and the difference in sperm parameters between groups.

Data collection followed the principles outlined in the Declaration of Helsinki. All men signed an informed consent agreeing to share their own anonymous information for future studies. The study was approved by our Hospital Ethical Committee (Prot. 2021—ESQLFDI).

### Statistical Methods

Distribution of data was tested with the Shapiro–Wilk test. Data are presented as medians (interquartile range; IQR) or frequencies (proportions). First, baseline clinical, laboratory parameters and semen characteristics were compared between the N and D groups with the Mann–Whitney test and the chi-square test. Second, univariable (UVA) and multivariable (MVA) logistic regression analyses tested the associations between study variables and oxidative stress markers.

Regarding antioxidant supplementation therapy, the Wilcoxon signed-rank test was used to assess potential differences in D-ROMs values and sperm parameters at 3 months follow-up assessment, compared to baseline, among both groups. At follow-up, semen parameters and D-ROMs were compared between groups with the Mann–Whitney test. Finally, univariable (UVA) and multivariable (MVA) logistic regression analyses tested the associations between study variables and >10% D-ROMs improvement after treatment [21].

Statistical analyses were performed using SPSS v.28 (IBM Corp., Armonk, NY, USA). All tests were two-sided, and statistical significance level was determined at *p* < 0.05.

## 3. Results

Table 1 details clinical, hormonal, and oxidative stress characteristics of the entire cohort, segregated according to shift work. Overall, median (IQR) age and BMI were 38 (34–41) years and 25.7 (23.7–27.8) kg/m^2^, respectively. Median TV was 18 (15–25) while median tT was 4.6 (3.7–7.2) ng/mL. Groups were similar in terms of clinical characteristics and recreational habits. Serum hormones were comparable among patients in the N and D groups. D-ROMs values were higher in group N than D [340 (295–375) U.CARR vs. 280 (241–305) U.CARR, *p* = 0.01]. Similarly, a higher proportion of men in the N group exhibited D-ROMs values suggestive for oxidative stress compared to those in group D (74.4% vs. 24.4%, *p* = 0.01).

In terms of semen parameters, sperm volume, concentration, and morphology were similar among groups. Progressive sperm motility was higher in the D group than N [46 (35–75)% vs. 35 (28–41)%, *p* = 0.02].

Table 2 reports the results of logistic regression models predicting D-ROMs > 300 in the whole cohort. In the univariable analysis (UVA), older age (OR 1.5, *p* = 0.01; 95% CI: 1.01–3.25), active smoking status (OR 1.2, *p* = 0.01; 95% CI: 1.09–3.46), and being a night worker (OR 2.3, *p* = 0.001; 95% CI: 1.15–5.38) were significantly associated with elevated oxidative stress levels (D-ROMs >300). Conversely, comorbidity burden (CCI ≥1), presence of varicocele, and serum total testosterone levels were not. Multivariable analysis (MVA) confirmed that night-shift work remained an independent predictor of D-ROMs > 300 (OR 2.1, *p* = 0.001; 95% CI: 1.12–4.36), after accounting for age and smoking status.

Table 3 reports oxidative stress results and sperm parameters before and after Drolessano treatment in both groups. At baseline, D-ROMs values were higher in group N than D [330 (299–365) U.CARR vs. 282 (245–303) U.CARR, *p* = 0.01]. Similarly, a higher proportion of men in the N group exhibited D-ROMs values suggestive for oxidative stress compared to those in group D (75% vs. 25%, *p* = 0.01). After Drolessano treatment, D-ROMs values improved in both groups (all *p* < 0.01 vs. baseline) with no difference between N and D men at follow-up. The magnitude of D-ROMs improvement was higher for men in the N group than those in the D one [mean change −58.5 (39.5) for N and −15.4 (15.3) for D, *p* = 0.001].

After Drolessano treatment, sperm concentration, progressive sperm motility and normal sperm morphology significantly improved in the N group (all *p* < 0.01). In the D group, only progressive sperm motility and normal sperm morphology significantly improved after treatment (all *p* < 0.01).

Table 4 reports logistic regression model predicting D-ROMs improvement >10% at follow up. Univariable analysis revealed that older age (OR 1.1, *p* = 0.01), active smoking status (OR 1.2, *p* = 0.01), along with N group (OR 3.4, *p* < 0.001), were all associated with D-ROMs improvement >10% after Drolessano treatment. Multivariable logistic regression analysis confirmed that N group (OR 3.2, *p* < 0.001) was an independent predictor of >10% D-ROMs improvement, after accounting for age and active smoking status (Cox & Snell R² = 0.4).

## 4. Discussion

The primary aim of our study was to investigate the impact of night-shift work on oxidative status in a cohort of men seeking fertility evaluation. Additionally, the study aimed to identify significant predictors of oxidative stress markers, including factors such as age, smoking status, and shift-work schedule. As a secondary aim, we conducted an exploratory pilot analysis to investigate the effectiveness of Drolessano, an oral antioxidant treatment, in reducing oxidative stress and improving semen parameters in night-shift workers compared to day-shift workers. Specifically, we sought to investigate whether the antioxidant intervention could potentially mitigate the increased oxidative burden typically associated with shift work, particularly for night-shift workers.

In our cohort, despite comparable clinical, hormonal, and lifestyle characteristics between day and night workers, night-shift workers exhibited higher levels of oxidative stress at baseline, confirmed by elevated D-ROMs values, compared to day-shift workers. Importantly, multivariable regression analysis confirmed night-shift work as an independent predictor of elevated oxidative stress, even after controlling for age and smoking status, two well-established contributors to oxidative stress. Regarding semen parameters, although sperm volume and concentration were similar at enrolment, night-shift workers had lower sperm motility compared to day-shift workers.

Collectively, these findings underscore the potential long-term health implications of shift work and highlight the need for preventive strategies targeting oxidative stress in this population.

After treatment with Drolessano, both groups showed significant improvements in oxidative stress markers, with the night-shift group demonstrating a more pronounced reduction in D-ROMs. Additionally, the night-shift group showed significant improvements in sperm concentration, progressive motility, and normal sperm morphology, while the day-shift group saw improvements primarily in motility and morphology. These preliminary findings are encouraging but must be interpreted with caution due to the lack of an untreated control group.

Our study was motivated by the growing concern surrounding the impact of oxidative stress on male reproductive health, particularly in populations exposed to shift work. Night-shift workers are known to exhibit elevated oxidative stress levels, which can adversely impact semen quality and overall reproductive function [24]. Indeed, in our cohort, night-shift workers exhibited elevated oxidative stress markers at baseline, indicating a higher level of oxidative damage compared to non-shift workers. Specifically, elevated D-ROMs values reflected increased systemic oxidative stress, aligning with previous studies that have associated oxidative imbalance with male infertility [25] and reduced total sperm count [26,27].

These findings are comparable with those observed in our pilot study, where antioxidant treatment was found to be associated with improvements in oxidative stress markers among night and day-shift workers. However, the night-shift group exhibited a more pronounced reduction in d-ROMs levels compared to the day-shift group. Research suggests that night-shift workers have an increased oxidative stress burden that could negatively affect sperm quality and overall reproductive health [14,28]. From a speculative standpoint, the marked improvement in oxidative stress markers in the night-shift group following antioxidant supplementation could represent a potential approach to mitigate the adverse effects of shift work on male fertility and general health. However, these results should be carefully interpreted taking in mind the pilot nature of this investigation in a limited number of patients and with the lack of a control group [29,30].

In literature, it is well-established that excessive oxidative stress can cause damage to sperm membranes, DNA, and mitochondria, all of which are essential for optimal sperm motility and overall fertility potential [31]. In our study, although sperm volume and concentration were similar between the two groups at enrolment, night-shift workers demonstrated considerably lower sperm motility compared to their day-shift counterparts. This suggests that the disruption of the circadian rhythm may have a more pronounced effect on sperm motility, even though other semen parameters, such as volume and concentration, were not as significantly impacted. In a recent meta-analysis, investigating the impact of night-shift work on male reproductive health, Viramgami et al. identified a reduction in sperm count in night-shift workers compared to controls [32], thereby supporting the hypothesis that circadian disruption may adversely affect spermatogenesis. Specifically, there appears to be a trend toward reduced sperm concentration and a lower proportion of morphologically normal and motile sperm among shift workers. These findings underscore a consistent association between night-shift work and impaired male reproductive function, particularly in terms of semen quality, likely mediated by mechanisms involving oxidative stress and circadian rhythm disruption.

Another study by Demirkol et al. aligns with previous findings, suggesting that shift work may have a detrimental effect on semen quality in men seeking infertility evaluation [13]. Specifically, shift workers had significantly higher rates of oligozoospermia compared to non-shift workers. Additionally, the mean normal morphology percentage was significantly lower in the shift worker group, suggesting that shift work may negatively impact sperm shape, which is crucial for fertility. Moreover, the study revealed that sleep quality, which is often disrupted by shift work, played a key role in these findings. Shift workers were more likely to report poor sleep, suggesting that circadian disruption commonly associated with shift work may impair sperm production and motility. These findings are further supported by the LIFE study, a prospective cohort investigation that examined the relationship between physical occupational exposures and semen quality, while controlling for health and socioeconomic factors [33]. The study identified a negative association between strenuous occupational activity and sperm count. Together, these findings reinforce the multifactorial nature of male infertility, wherein occupational, physiological, and lifestyle factors converge, particularly in populations exposed to circadian rhythm disruption, such as night-shift workers.

In terms of serum hormones, previous studies have shown that shift workers had lower sperm counts but marginally higher serum testosterone levels compared to controls [32]; however, the low quality of these results precludes a general interpretation. In this study we could not find a difference in serum hormones between the N and D groups.

In our study, we identified night-shift work, older age, and active smoking as significant predictors of a reduction in semen quality, with night-shift status being the most prominent factor. This finding is consistent with existing research, which has consistently demonstrated that night-shift work, in combination with older age and smoking, is closely associated with increased oxidative stress [13]. What is particularly noteworthy in both studies is that, despite the higher prevalence of smoking and older age in the non-shift-worker group, factors that are widely recognized as detrimental to semen quality, shift workers nevertheless demonstrated inferior semen quality overall. This suggests that the detrimental effects of shift work, especially the disruption of circadian rhythms, may have a more substantial impact on semen quality than lifestyle factors like smoking or age alone.

While antioxidants play a vital role in counteracting oxidative stress, caution is necessary to avoid the so-called “antioxidant paradox.” This phenomenon refers to the potential negative effects of excessive antioxidant supplementation, which can disrupt the body’s delicate redox balance [34]. Some degree of oxidative stress is essential for various physiological processes, including sperm maturation, capacitation, and fertilization. A complete elimination of oxidative stress might interfere with these natural processes, potentially impairing sperm function and fertility. Overuse of antioxidants can inhibit natural cellular signaling, affecting sperm motility and DNA integrity. This underscores the importance of targeted antioxidant therapy, with carefully considered dosages tailored to individual needs. As research in this area continues to evolve, determining the appropriate levels of antioxidant supplementation will be crucial to ensuring its efficacy and safety in treating male infertility. Furthermore, more studies are needed to establish standardized treatment protocols, which may involve both the type and timing of antioxidant use for maximum benefit.

Our study is not devoid of limitations. First, the relatively small sample size and single-centre design inherently restrict the generalizability of our findings. Larger, multicentric studies are necessary to confirm the reproducibility and external validity of our results across broader and more diverse populations. Second, and most critically, the absence of an untreated (non-intervention) control group substantially limits the interpretability of our findings. Without such a comparator, we cannot determine whether the observed changes in oxidative stress markers and semen parameters are a direct result of the antioxidant treatment, or rather reflect natural temporal fluctuation, placebo effects, or other nonspecific factors. This is a major limitation that precludes any definitive conclusions about causality. Third, despite the homogeneous evaluation by a single experienced urologist and the use of standardized protocols, the retrospective nature of the study limits control over potential confounders. Unmeasured variables, including diet, sleep patterns, physical activity, psychological stress, circadian misalignment, and environmental exposures, may have influenced oxidative stress and semen quality. Notwithstanding these limitations, this study serves as a preliminary exploration, and we are currently planning a prospective, randomized, placebo-controlled trial to rigorously assess the effects of antioxidant supplementation on oxidative stress and semen parameters in both night- and day-shift workers.

## 5. Conclusions

Night-shift workers showed significantly higher levels of oxidative stress compared to day workers, with particularly elevated D-ROMs values, indicating a greater oxidative imbalance. This difference likely stems from the biological impact of circadian rhythm disruption. These findings support the fact that night workers are biologically more susceptible to oxidative stress. We showed preliminary results indicating that, following antioxidant treatment, both groups had improvements in oxidative markers; however, the reduction in D-ROMs was more pronounced among night-shift workers, suggesting a stronger compensatory response. Therefore, future prospective, randomized studies are needed to more comprehensively investigate the role of antioxidant treatment against oxidative stress in shift workers.

## Figures and Tables

**Table 1:** Demographic characteristics of the whole cohort of patients (N = 96).

	Overall	Night Workers	Day Workers	*p*-Value *
No. of individuals	96	47 (48.9%)	49 (51.1%)	
Age (years)				0.5
Median (IQR)	38 (34–41)	38 (35–41)	37 (33–40)	
Range	25–45	25–45	25–44	
BMI (kg/m^2^)				0.6
Median (IQR)	25.7 (23.7–27.8)	25.5 (23.8–26.3)	25.6 (23.6–28.7)	
Range	18.9–29.9	18.9–29.5	20.9–29.9	
CCI (score)				0.8
Median (IQR)	0.0 (0.0)	0.0 (0.0)	0.0 (0.0)	
Mean (SD)	0.1 (0.3)	0.1 (0.2)	0.1 (0.2)	
Range	0–4	0–3	0–4	
Current smoking status [No. (%)]	44 (45.8)	21 (44.6)	23 (46.9)	0.7
Mean TV (Prader’s estimation)				0.9
Median (IQR)	18 (15–25)	18 (15–25)	18 (14–25)	
Range	5–25	5–25	8–25	
Varicocele [No. (%)]	15 (15.6)	7 (14.8)	8 (16.3)	0.8
tT (ng/mL)				0.4
Median (IQR)	4.6 (3.7–7.2)	4.5 (3.6–7.0)	4.6 (3.9–7.9)	
Range	3.5–12.8	3.6–12.8	3.5–12.7	
FSH (mUI/mL)				0.3
Median (IQR)	6.5 (3.3–9.6)	6.4 (3.1–9.1)	6.5 (3.4–10.1)	
Range	2.4–10.8	2.4–9.7	2.3–10.8	
LH (mUI/mL)				0.8
Median (IQR)	5.7 (3.3–8.1)	5.5 (3.7–8.0)	5.6 (3.5–8.2)	
Range	1.8–12.1	1.8–12.1	3.1–12.0	
Prolactin (ng/mL)				0.8
Median (IQR)	8.4 (5.6–11.9)	8.1 (5.1–11.6)	8.3 (5.7–11.9)	
Range	1.9–24.3	1.9–20.1	2.6–24.3	
Metabolites-D-ROMs (U.CARR)				0.01
Median (IQR)	302 (258–355)	340 (295–375)	280 (241–305)	
Range	203–405	260–405	203–330	
D-ROMs > 300 [No. (%)]	47 (48.9)	35 (74.4)	12 (24.4)	<0.01
Semen volume (mL)				0.7
Median (IQR)	3.0 (2.0–4.5)	3.0 (2.0–4.4)	3.0 (2.1–4.0)	
Range	1.0–10.0	1.0–9.0	1.0–10.0	
Sperm concentration (×10^6^/mL)				0.2
Median (IQR)	35.7 (18.9–55.2)	33.8 (18.3–49.9)	41.1 (20.2–58.7)	
Range	9.1–90.2	11.2–73.3	9.1–90.2	
Progressive sperm motility (%)				0.02
Median (IQR)	41 (30–68)	35 (28–41)	46 (35–75)	
Range	5–90	5–71	12–90	
Normal sperm morphology (%)				0.07
Median (IQR)	3 (2–6)	2 (1–6)	3 (2–4)	
Range	0–10	0–4	0–10	

Keys: BMI = body mass index; CCI = Charlson Comorbidity Index; TV = testicular volume; tT = total Testosterone; FSH = follicle-stimulating hormone; LH = luteinizing hormone; d-ROMs = derivatives of reactive oxidative metabolites; * *p* value according to the Mann–Whitney test and chi-square test, as indicated.

**Table 2:** Logistic regression models predicting D-ROMs >300 in the whole cohort.

	UVA Model		MVA Model	
	OR, *p*-Value	95% CI	OR, *p*-Value	95% CI
Age	1.5, 0.01	1.01–3.25	1.2, 0.02	1.11–3.27
CCI ≥ 1	1.2, 0.6	0.46–3.37		
Smoking status	1.2, 0.01	1.09–3.46	1.1, 0.03	1.09–2.21
Varicocele	1.1, 0.2	0.98–1.51		
Serum tT	0.8; 0.4	0.72–1.19		
Night worker	2.3, 0.001	1.15–5.38	2.1; 0.001	1.12–4.36

Keys: UVA = Univariate model; MVA = Multivariate model, CCI = Charlson Comorbidity Index; tT = total Testosterone.

**Table 3:** Seminal and oxidative stress parameters before and after treatment in the pilot study (N = 40).

	Night Workers	Day Workers	*p*-Value
Reactive Oxygen			
Metabolites-D-ROMs (U.CARR)			
Baseline			0.01
Median (IQR)	330 (299–365)	282 (245–303)	
Range	260–403	203–320	
D-ROMs > 300 [No. (%)]	15 (75.0)	5 (25.0)	<0.01
Follow-up			0.1
Median (IQR)	270 (253–289) §	260 (234–295) §	
Range	245–305	204–301	
D-ROMs > 300 [No. (%)]	3 (15.0)	4 (20.0)	0.6
Change from baseline			0.001
Mean (SD)	−58.5 (39.5)	−15.4 (15.3)	
Range	−126–5.0	−44.0–12.0	
Semen volume (mL)			
Baseline			0.8
Median (IQR)	3.0 (2.0–4.5)	3.0 (2.0–4.0)	
Range	1.0–10.0	1.0–9.0	
Follow-up			0.8
Median (IQR)	3.0 (2.0–4.0)	3.0 (2.0–4.0)	
Range	1.0–11.0	1.0–10.0	
Sperm concentration (×10^6^/mL)			
Baseline			0.1
Median (IQR)	31.9 (18.1–49.3)	40.1 (20.6–58.6)	
Range	10.9–70.3	9.1–90.2	
Follow-up			0.5
Median (IQR)	48.1 (29.1–60.3) §	47.2 (26.9–59.9)	
Range	14.9–101.7	13.4–99.8	
Progressive sperm motility (%)			
Baseline			0.02
Median (IQR)	35 (28–41)	46 (35–75)	
Range	5–71	12–90	
Follow-up			0.1
Median (IQR)	48 (37–71) §	52 (38–77) §	
Range	20–89	12–98	
Normal sperm morphology (%)			
Baseline			0.6
Median (IQR)	2 (1–6)	2 (1–6)	
Range	0–10	0–10	
Follow-up			0.6
Median (IQR)	4 (1–6) §	4 (1–6) §	
Range	2–10	2–10	

Keys: d-ROMs = derivatives of reactive oxidative metabolites. *p* value according to the Mann–Whitney test, as indicated. § *p* < 0.01 vs. baseline. *p* value according to the Wilcoxon signed-rank test.

**Table 4:** Logistic regression models predicting >10% D-ROMs improvement in the pilot study.

	UVA Model			MVA Model		
	OR	*p*-Value	95% CI	OR	*p*-Value	95% CI
Age	1.1	0.01	1.09–2.95	1.1	0.03	1.08–2.92
CCI	1.0	0.5	0.87–3.12			
TV	1.1	0.3	0.91–2.76			
Total testosterone	0.8	0.2	0.68–1.15			
Active smoking status	1.2	0.01	1.09–2.45	1.2	0.02	1.10–3.21
Night work	3.4	<0.001	1.97–7.99	3.2	<0.001	2.51–9.91

Keys: UVA = Univariate model; MVA = Multivariate model, CCI = Charlson Comorbidity Index; TV = testicular volume.

## Data Availability

Data is contained within the article.
